# Screening Specific Biomarkers of Herbs Using a Metabolomics Approach: A Case Study of *Panax ginseng*

**DOI:** 10.1038/s41598-017-04712-7

**Published:** 2017-07-04

**Authors:** Hong-ping Wang, Yan Liu, Chang Chen, Hong-bin Xiao

**Affiliations:** 1 Beijing University of Chinese Medicine, Beijing, China; 2 Institute of Chinese Materia Medica, China Academy of Chinese Medical Sciences, Beijing, 100700 China; 3 Shihezi University, Shihezi, China

## Abstract

Medicinal herbs belonging to the same genus are always easily confused due to their extremely similar morphology and metabolites. Previously, to differentiate them, inherently specific biomarkers were screened out via intuitive comparison of their metabolite profiles. Unfortunately, the selected biomarkers have worked only partially. Most significant specific biomarkers have been neglected. Herein, a novel method for screening specific biomarkers of medicinal herbs using a metabolomics technique was developed. Firstly, the profiles of a group of easily confused herbs belonging to the same genus were analyzed by ultra-high performance liquid chromatography coupled with high-resolution mass spectrometry to detect all components, including low-response metabolites. Then, all components were compared between the different samples, and specific biomarkers were extracted by the metabolomics techniques of alignment, normalization, defining the sample sets, filtering by frequency and Venn diagram analysis with Mass Profiler Professional (MPP) software. Thirdly, the correlations of these biomarkers were investigated via partial correlational analysis to obtain the most representative specific biomarkers. As an example, selection of specific biomarkers for ginseng (*Panax ginseng*) was performed, and three specific biomarkers including chikusetsusaponin IVa, ginsenoside Rf and ginsenoside Rc were finally selected and verified as the most representative specific biomarkers of *Panax ginseng*.

## Introduction

Metabolites are the final products of cellular regulation, and their types as well as quantities are deemed the final response of a biological system to changes in genes or the environment. It has been estimated that there are more than 200,000 metabolites in the plant kingdom, including primary metabolites used to maintain plant life and secondary metabolites used to protect plants against biotic and abiotic stress. The metabolites in plants play an important role in their growth, and the composition of different organisms directly correlates with their properties. In most Asian countries, many plants, which are usually called medicinal herbs, are always used to cure certain diseases due to their treatment efficacy for many diseases, and the metabolites are responsible for the curative effects. Usually, the metabolites in medicinal herbs belonging to different genera are greatly different, and common metabolites are rare. However, medicinal herbs in the same genus always have great similarity not only in morphology and gene DNA sequences but also in their chemical metabolites because of their similar metabolic pathways. This can easily lead to confusion in their clinical use and raise questions regarding the clinical safety of medications.

It is well known that the formation and distribution of secondary metabolites are always species specific, and the metabolites in medicinal herbs belonging to different genera are always greatly different. However, the metabolites in medicinal herbs belonging to the same genus are greatly similar, and each medicinal herb species contains its own specific chemical metabolites, which can be selected as specific biomarkers to differentiate a particular species from other similar herbs. Until now, the selection of a specific biomarker from hundreds of metabolites in medicinal herbs has been a crucial problem. Some researchers have compared the metabolite profiles of crude extracts of similar medicinal herbs in a non-targeted manner using the premise of desirable chromatographic separation, and the specific metabolites were intuitively chosen and then analyzed by mass spectrometry (MS)^1^ or nuclear magnetic resonance (NMR)^2^. With the help of MS^3, 4^ and tandem mass spectrometry (MS/MS) spectral databases^5–8^ and NMR chemical shift databases^9, 10^, some known and unknown specific metabolites can be identified. However, the estimation of the chemical structures of unknown metabolites in metabolite profile data continues to present challenges^11^ because of a lack of reference standards. In fact, the results are not particularly accurate, and the selected specific biomarkers were incomplete. Most of the specific biomarkers have not been analyzed due to samples containing many overlapping signals; in addition, the most intense signals were usually picked out to represent metabolites of interest, and the less intense signals were always neglected. Moreover, whether the selected specific biomarkers are typical cannot be confirmed. Therefore, it is still a great challenge to analyze their metabolite profiles and to select specific biomarkers.

In our experiment, we solved this problem using metabolomics technology, which studies the body from a holistic perspective in an extremely duplicable system and now plays a significant role in many biological fields^12–18^. Especially in the plant field, metabolomics can systematically analyze the overall metabolites and yield specific biomarkers contributing to classification and making the effective screening of crucial specific metabolites a reality. Mass spectrometry (MS)-based metabolomics is well suited for reliably handling high-throughput samples with respect to both technical accuracy and the identification and quantification of low-molecular-weight metabolites. The advantages of MS-based metabolomics are best realized when coupled to liquid chromatography (LC). In particular, ultra-high performance liquid chromatography coupled with quadrupole time-of-flight mass spectrometry (UPLC-Q-TOF MS) has been widely used for metabolomics studies because of its high sensitivity, high accuracy and high resolution, which indicates that even low-response metabolites can be collected and identified. In addition, the high resolution of MS and MS/MS information on metabolites is helpful for metabolite identification. Generally, there is more than one specific biomarker in one herb, and it is inadvisable to use all of the specific biomarkers to differentiate one herb from others due to some reference standards being unavailable or to avoid making the experiment much more complicated. In our experiment, we analyzed the relationships of specific biomarkers using the correlational analysis strategy, and the most representative specific metabolites were selected as the final specific biomarkers.

In this study, we use ginseng (*Panax ginseng)* as an example to demonstrate how to screen for specific biomarkers using a metabolomics approach. *Panax ginseng* is well known as the lord or king of herbs and is now widely used not only in Asian countries but also in many western countries. Obviously, medicinal herbs not belonging to the *Panax* genus have hardly any similarity in metabolites, morphology or gene DNA sequence with *Panax ginseng*. Therefore, it is easy to differentiate *Panax ginseng* from them. However, medicinal herbs belonging to the same genus with *Panax ginseng* share great similarity in many aspects, especially in their chemical metabolites because of their similar metabolic pathways. The three most common herbs in *Panax* genus, including *Panax notoginseng*, *Panax quinquefolium* and *Panax japlcus var*, have many metabolites in common with *Panax ginseng*, and even their metabolite profiles are extremely similar. However, these four herbs cannot replace each other in clinical use. To avoid confusion with the other three herbs, the specific biomarkers of *Panax ginseng* were selected from their extremely duplicable metabolite profiles using metabolomics technology. Finally, three metabolites, including chikusetsusaponin IVa (Ch-IVa), ginsenoside Rf and ginsenoside Rc, were selected as the most representative specific biomarkers of *Panax ginseng* via correlational analysis; moreover, they can obviously differentiate *Panax ginseng* from the others.

## Results and Discussion

### Method validation and metabolite profiling process

The metabolome content of *Panax ginseng* (A), *Panax notoginseng* (B), *Panax quinquefolium* (C) and *Panax japlcus var* (D) was analyzed by UPLC-Q-TOF MS. Metabolites were extracted with 70% aqueous MeOH, separated on a C_18_ column, and analyzed by MS in the negative mode. For a method validation study, 20 μL samples from each of the four groups were pooled to obtain a quality control (QC) specimen, and the acquisition of the QC specimen was the same as that of the samples. A number of consecutive injections of the QC sample were made to obtain a stable Q-TOF MS system before experimental data acquisition, and the acquisition of data for samples was then started. A QC sample was analyzed every 6 samples throughout the whole analysis procedure.

For the QC sample, five characteristic ions (*m/z* 931.5266 with retention time 4.58 min; *m/z* 799.4844 with retention time 6.76 min; *m/z* 1077.5850 with retention time 7.52 min; *m/z* 945.5423 with retention time 8.07 min; *m/z* 793.4374 with retention time 9.52 min) were chosen to examine the shifts in retention time, *m/z* and peak area to assess the stability of the system. The results (shown in Table S3) showed that the deviations of *m/z* for each ion were less than 2.63 × 10^−6^, and the relative standard deviations (*RSDs*) of retention time, peak area and peak intensity for each peak were less than 0.13%, 8.54% and 3.45%, respectively. As we know, the excellent stability and repeatability of an analysis system can yield reasonable data, which can be further processed to obtain credible results. The data above demonstrated that the system had excellent stability and repeatability during the analysis procedure.

**Figure 1:**
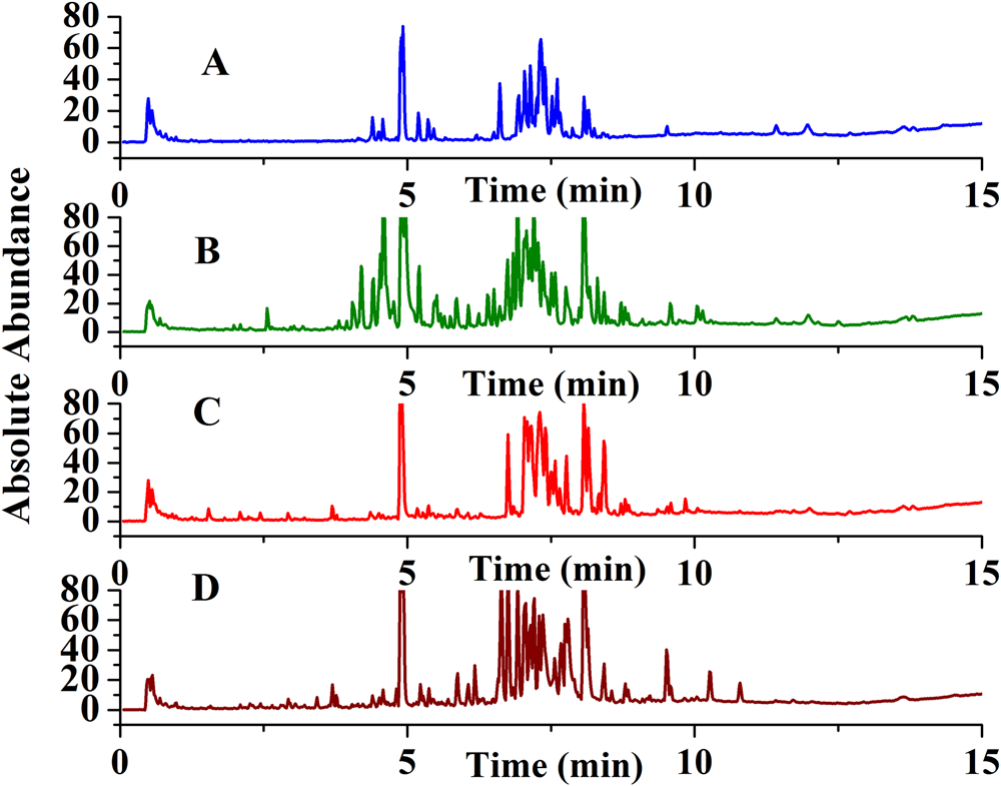
Metabolite profiling of the medicinal herbs in negative-ion mode: *Panax ginseng* (**A**); *Panax notoginseng*; (**B**); *Panax quinquefolium* (**C**); and *Panax japlcus var* (**D**).

**Figure 2:**
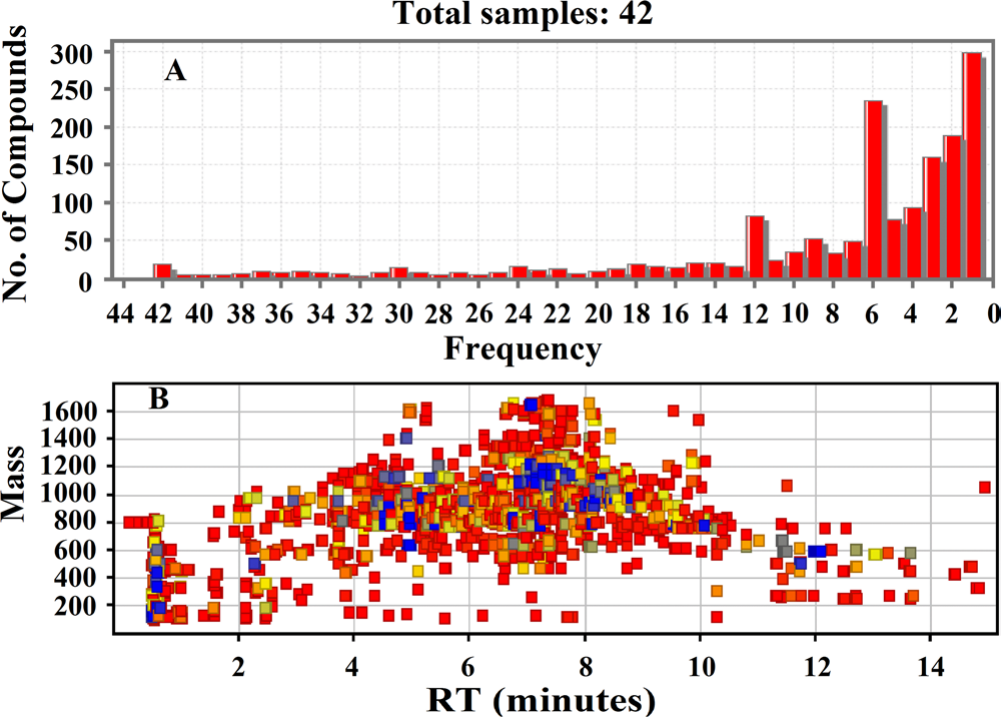
The overall situation of aligned metabolites in 42 samples (**A**) and the mass-retention curve of aligned metabolites (the lower frequency metabolites are marked by red color, while the higher frequency metabolites are marked by blue color).

The obtained typical total ion chromatograms (TICs) for the four herbs seemed closely similar (shown in Fig. 1). Most major peaks found in the TICs appeared in almost all four herbs. The UPLC-Q-TOF MS data for each herb were further processed with Mass Hunter software (version B.06.00, Agilent, America) to recognize the ion peaks and extract the chemical metabolites with the help of the “*find compounds by molecular feature*” function. The extracted metabolites were exported as.cef files, and these files were then imported into Mass Profiler Professional (MPP) software (version B.12.00, Agilent) for further analysis including alignment, normalization, defining the sample sets, filtering by frequency and Venn diagram analysis. As a result, a total of 1634 metabolites were aligned among 42 samples (shown in Fig. 2). From Fig. 2A, we found that most of the metabolites presented low frequency, and many of the metabolites presented only once or twice, which was confirmed by the mass-retention curve of metabolites after alignment (shown in Fig. 2B). The lower frequency metabolites were marked by red color, while the higher frequency metabolites were marked by blue color; the red ones account for most of the metabolites. These lower frequency metabolites will be excluded by setting proper filtering parameters to generate higher quality data, leading to a much more meaningful analysis. In our experiment, the lower frequency metabolites were filtered according to their frequency, and the metabolites that appeared in 100% of samples in at least one group were retained.

**Figure 3:**
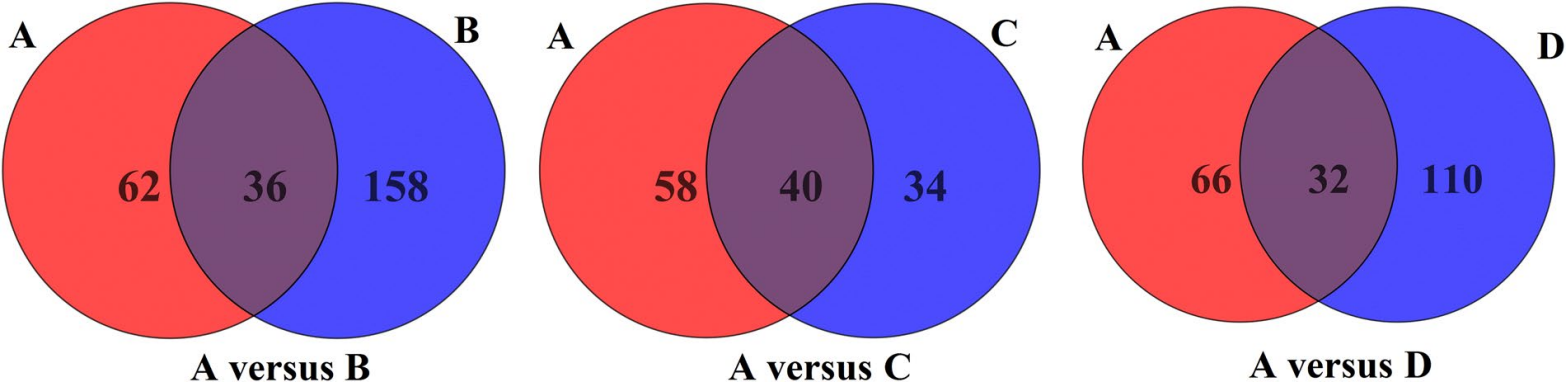
Venn diagrams of pairwise analyses: **A versus B** (*Panax ginseng* versus *Panax notoginseng*), **A versus C** (*Panax ginseng* versus *Panax quinquefolium*) and **A versus D** (*Panax ginseng* versus *Panax japlcus var*).

After filtering, there were 98 metabolites in *Panax ginseng* (A), 194 metabolites in *Panax notoginseng* (B), 74 metabolites in *Panax quinquefolium* (C) and 142 metabolites in *Panax japlcus var* (D). To reveal the specific metabolites in *Panax ginseng* (A), pairwise analysis, including A versus B, A versus C, and A versus D, was performed using a Venn diagram (shown in Fig. 3). The results indicated that 62 specific metabolites were found in A versus B, 58 specific metabolites were found in A versus C, and 66 specific metabolites were found in A versus D. The respectively obtained specific metabolites in *Panax ginseng* were checked manually, and we found that they contained redundant signals caused by different isotopes, in-source fragmentation, and HCOO^−^ adducts. To produce a matrix containing fewer biased and redundant data, the redundant signals were manually removed.

Finally, the highly reproducible and non-redundant metabolite signals were obtained as follows: 26 specific metabolites were retained in A versus B (shown in Table S4), 23 specific metabolites were retained in A versus C (shown in Table S5), and 30 specific metabolites were retained in A versus D (shown in Table S6). We deemed these retained metabolites the specific and effective metabolites of *Panax ginseng*. The respectively obtained data matrix was then used for further correlational analysis. In addition, due to triterpenoid saponins being the major effective metabolites in *Panax ginseng*, we hoped the final indexes would be triterpenoid saponins.

### Specific metabolite identification

**Figure 4:**
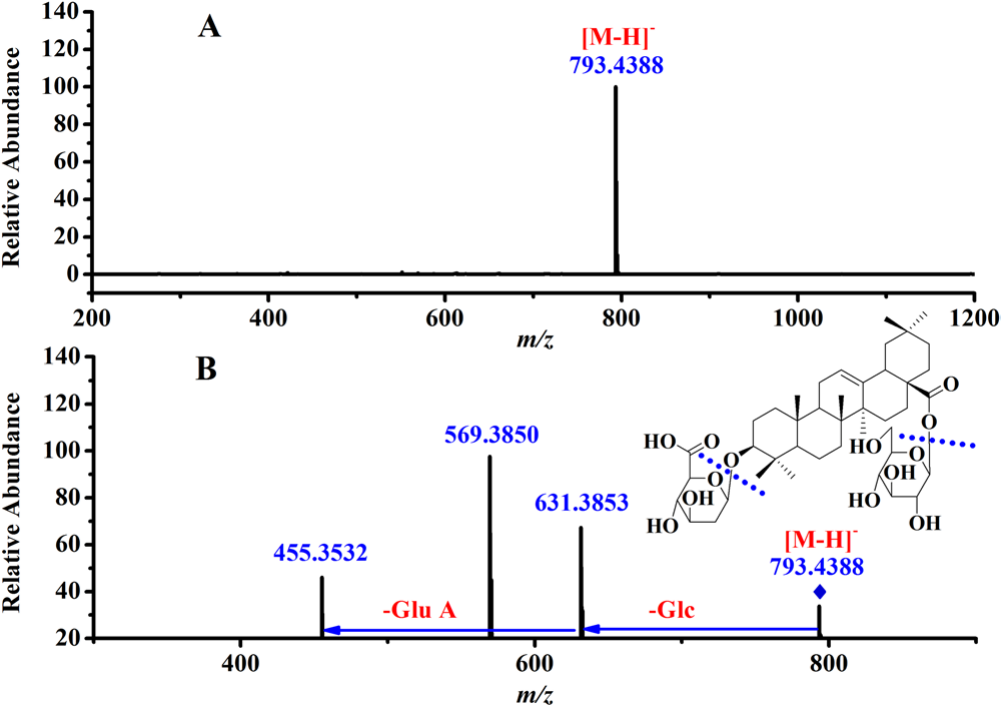
MS spectrum of metabolite **R23** (**A**) and MS/MS spectrum of metabolite **R23** (**B**).

The specific metabolites for A versus B, A versus C and A versus D have been successfully obtained, and their accurate mass-to-charge ratio (*m/z*) values as well as their retention times have been manually recorded. To facilitate their identification, the fragmentation ion for each of the corresponding accurate *m/z* values was obtained by running the analysis under targeted MS/MS mode. Due to triterpenoid saponins being the major metabolites and responsible for the curative effects of the four herbs, the identification of the triterpenoid saponins seemed to be much more important. Actually, the diagnostic ions and fragmentation pathways of the triterpenoid saponins have been previously reported^19–21^, and we used them to deduce the known and unknown triterpenoid saponin metabolites in our experiment. Metabolite **R23** eluted at 8.09 min gave the precursor ion at *m/z* 793.4388 (shown in Fig. 4A), indicating that its molecular formula was C_42_H_66_O_14_. The MS/MS spectrum showed that the aglycone ion was at *m/z* 455.3532, suggesting that it was oleanolic acid-type ginsenoside (shown in Fig. 4B). The fragmentation ions observed at *m/z* 631.3853 and 455.3532 suggested that Glc and Glu A were successively eliminated from the [M-H]^−^ ion. Thus, metabolite **R23** was tentatively assigned as chikusetsusaponin IVa (Ch-IVa). Metabolite **X21** eluted at 6.62 min, and the [M-H]^−^ ion was observed at *m/z* 799.4850, which indicated that the molecular formula was C_42_H_72_O_14_. The MS/MS spectrum showed that the aglycone ion was at *m/z* 475.3825, suggesting that it was PPT-type ginsenoside. The fragmentation ions at *m/z* 637.4254 and 475.3825 indicate that Glc and Glc were successively eliminated from the [M-H]^−^ ion. Thus, metabolite **X21** was deduced as ginsenoside Rf. In the same way, five other triterpenoid saponins including ginsenoside Rc (**Z11**), Ia (**X20**), Re_4_ (**Z1**), malonyl-Rc (Ma-Rc, **R20**/**Z20**) and malonyl-Rb_3_ (Ma-Rb_3_, **R21**/**X15**) were tentatively identified. To determine the structure of the metabolite peak *m/z* 931.5255 (**X1**), an MS/MS experiment was performed, and fragmentation ions were observed at *m/z* 799.4899, 637.4287 and 475.3819, indicating that Xyl, Glc and Glc were successively eliminated from the [M-H]^−^ ion. The fragmentation pathway is similar to that of notoginsenoside R_1_^19, 20^; thus, metabolite **X1** was tentatively identified as a notoginsenoside R_1_ isomer (Noto-R_1_-iso). Metabolites **X7**, **X8** and **X9**, having the same precursor ion at *m/z* 841.4961, eluted at 5.20 min, 5.46 min and 6.82 min, respectively. In their MS/MS spectrum, fragmentation ions were observed at *m/z* 799.4901, 637.4284 and 475.3797, indicating that Ac, Glc and Glc were successively eliminated from the [M-H]^−^ ion. After losing the Ac group, their fragmentation pathway was the same as that of ginsenoside Rf or Rg_1_. Furthermore, ginsenoside Rg_1_ always eluted prior to ginsenoside Rf^19–21^; thus, **X7**, **X8** and **X9** were tentatively assigned as Acetyl-Rg_1_, Acetyl-Rg_1_ isomer (Ace-Rg_1_-iso) and Acetyl-Rf, respectively. All of the fragmentation ions of the identified metabolites are shown in Table S7.

To confirm the accurateness of the identification, three metabolites including Ch-IVa (**R23**), ginsenoside Rf (**X21**) and Rc (**Z11**) were analyzed using the same profiling procedure as used for the extracts. By comparing the *m/z* values, retention time, and the fragmentation pathway with those of their reference standards, they were confirmed unambiguously. However, those metabolites for which reference standards are commercially unavailable are still tentatively deduced, and their structures could not be unambiguously identified.

### Correlational analysis of the specific biomarkers

**Figure 5:**
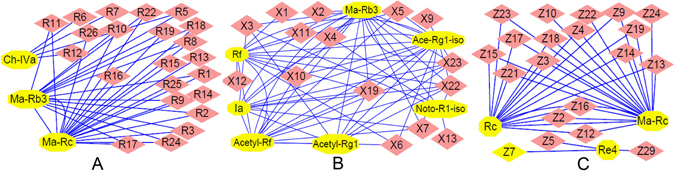
Correlational analysis of the triterpenoid saponins with other specific biomarkers for *Panax ginseng* versus *Panax notoginseng* (**A**), *Panax ginseng* versus *Panax quinquefolium* (**B**) and *Panax ginseng* versus *Panax japlcus var* (**C**).

As is well known, the metabolites in a definite herb present inherent functional relationships or proportional relations, and we can deduce the changes to other relevant metabolites (for which commercial standards are unavailable) according to the changes of the crucial metabolites (for which commercial standards are available), which have the most relevant relationship with others. In our experiment, the crucial metabolites were screened via correlational analysis, and a positive correlation, a relationship between variables in which variables move in tandem and one variable increases as the other variable increases, was determined. The peak areas of the specific metabolites obtained via the integral participated in the positive correlational analysis. Pearson’s partial correlational analysis was performed, and the correlation networks were presented in Cytoscape software (shown in Fig. 5).

A versus B: As shown in Table S4, 26 metabolites were deemed the specific biomarker, and three of the metabolites were ginsenosides, which are regarded as the bioactive metabolites of *Panax ginseng*. The correlation network of the three ginsenosides and the other 23 metabolites is shown in Fig. 5A. From Fig. 5A, we found that the peak area of Ch-IVa had a positive correlation with the peak areas of **R5**, **R6**, **R7** and **R12**. However, the peak area of Ma-Rc almost has a positive correlation with the remaining metabolites, including Ma-Rb_3_. These results indicated that as the content of Ma-Rc and Ch-IVa increased, the content of the other metabolites increased. Therefore, Ma-Rc and Ch-IVa were deemed the representative metabolites. However, the acyl bond of malonyl-ginsenosides is extremely unstable, and it is easily hydrolyzed under conditions of acid, alkali, hot water or hot methanol. Moreover, because the relative abundance of Ch-IVa could be above 30%, much higher than that of Ma-Rc and other metabolites (the relative abundance of each metabolite was lower than 10%), the final specific biomarker for A versus B was Ch-IVa.

A versus C: As shown in Table S5, there were 7 ginsenosides among 23 specific metabolites, and the correlational analysis between the 7 ginsenosides and the other metabolites is shown in Fig. 5B. From Fig. 5B, we found that the peak area of ginsenoside Rf had a strong positive correlation with 11 metabolites, including **X1**, **X5**, **X6**, **X8–X12**, **X19**, **X22** and **X23**. The peak area of Ma-Rb_3_ almost has a positive correlation with the remaining metabolites. These results suggested that as the content of ginsenoside Rf and Ma-Rb_3_ increased, the content of the other metabolites increased. Thus, ginsenoside Rf and Ma-Rb_3_ were deemed the representative metabolites. Similarly, because Ma-Rb_3_ is unstable and the relative abundance of ginsenoside Rf could be above 30%, which is much higher than that of Ma-Rb_3_ and other metabolites (the relative abundance of each metabolite was lower than 3%), the final specific biomarker for A versus C was ginsenoside Rf.

A versus D: As shown in Table S6, there were 3 ginsenosides among 30 specific metabolites, and the correlations between the 3 ginsenosides and the other metabolites were analyzed (shown in Fig. 5C). From Fig. 5C, we found that the peak area of ginsenoside Rc had a strong positive correlation with the peak areas of 15 metabolites, including **Z2–Z4**, **Z9**, **Z12–Z19**, **Z21**, **Z23** and Ma-Rc. This result indicated that their content would increase as the content of ginsenoside Rc increases. Although the peak area of ginsenoside Re_4_ had a positive correlation with the other partial metabolites, including **Z5**, **Z7** and **Z29**, the relative abundance of ginsenoside Re_4_ was lower than 10%, which is much lower than that of ginsenoside Rc. Therefore, the final specific biomarker for A versus D was ginsenoside Rc.

### Verification of the specific biomarkers

To verify the specific biomarkers, additional samples (shown in Table S2) were collected and analyzed. All samples in Table S1 and Table S2 were included in the verification experiment.

For A versus B, Ch-IVa was selected as the specific biomarker of *Panax ginseng* according to the above data analysis. The distribution of Ch-IVa in the metabolite profiles of *Panax ginseng* and *Panax notoginseng* was observed (shown in Fig. S1). From Fig. S1, we found that the peak of Ch-IVa in both samples overlapped with other compounds, and it was difficult to intuitively observe if it was absent or present. Then, we used extracted ion chromatography (EIC) for Ch-IVa from the metabolite profiles of *Panax ginseng* and *Panax notoginseng* and integrated its peak areas. The recorded peak areas of Ch-IVa are shown in Table S8. From Table S8, we found that the *RSD* of the peak areas of Ch-IVa in *Panax ginseng* was 15.83%, indicating that the content of Ch-IVa in different *Panax ginseng* samples was relatively stable. However, the peak areas of Ch-IVa in *Panax notoginseng* were extremely small, indicating that only a trace amount of Ch-IVa existed in *Panax notoginseng*, which was confirmed by its MS spectrum (shown in Fig. S2). From Fig. S2, we found that the absolute abundance of the precursor ion of Ch-IVa in *Panax notoginseng* was lower than 5000 counts, and metabolite Ch-IVa was filtered in the process of data analysis. As a matter of course, Ch-IVa was deemed the representative and specific biomarker of *Panax ginseng* for A versus B.

For A versus C, ginsenoside Rf was selected as the specific biomarker of *Panax ginseng* according to the above data analysis. The distribution of Rf in the metabolite profiles of *Panax ginseng* and *Panax quinquefolium* was observed (shown in Fig. S3). From Fig. S3, we found that the peak of Rf was present in *Panax ginseng* and absent from *Panax quinquefolium*. After extracting ion chromatography for Rf from the metabolite profiles of *Panax ginseng* and *Panax quinquefolium* and integrating its peak areas, we found that the *RSD* of the peak areas of Rf in *Panax ginseng* was 14.80% (shown in Table S9), indicating that the content of Rf in different *Panax ginseng* samples was relatively stable. Similarly, the extremely small peak areas of Rf in *Panax quinquefolium* suggest only trace amounts of Rf in *Panax quinquefolium*. The absolute abundance of the precursor ion of Rf in *Panax quinquefolium* was lower than 5000 counts (shown in Fig. S4), and metabolite Rf was filtered in the process of data analysis. Finally, ginsenoside Rf was deemed the representative and specific biomarker of *Panax ginseng* for A versus C.

For A versus D, ginsenoside Rc was selected as the specific biomarker of *Panax ginseng* according to the above data analysis. The distribution of Rc in the metabolite profiles of *Panax ginseng* and *Panax japlcus var* was observed (shown in Fig. S5). From Fig. S5, we found that the peak of Rc in both samples overlapped with other metabolites. After extracting ion chromatography of Rc from the metabolite profiles of *Panax ginseng* and *Panax japlcus var* and integrating its peak areas, we found that the *RSD* of the peak areas of Rc in *Panax ginseng* was 19.31% (shown in Table S10), indicating that the content of Rc in different *Panax ginseng* samples was relatively stable. In the same way, the extremely small peak areas of Rc in *Panax japlcus var* suggest only trace amounts of Rc in *Panax japlcus var*. In addition, the absolute abundance of the precursor ion of Rc in *Panax japlcus var* was lower than 5000 counts (shown in Fig. S6), and metabolite Rc was filtered in the process of data analysis. It was feasible to select ginsenoside Rc as the representative and specific biomarker of *Panax ginseng* for A versus D.

**Figure 6:**
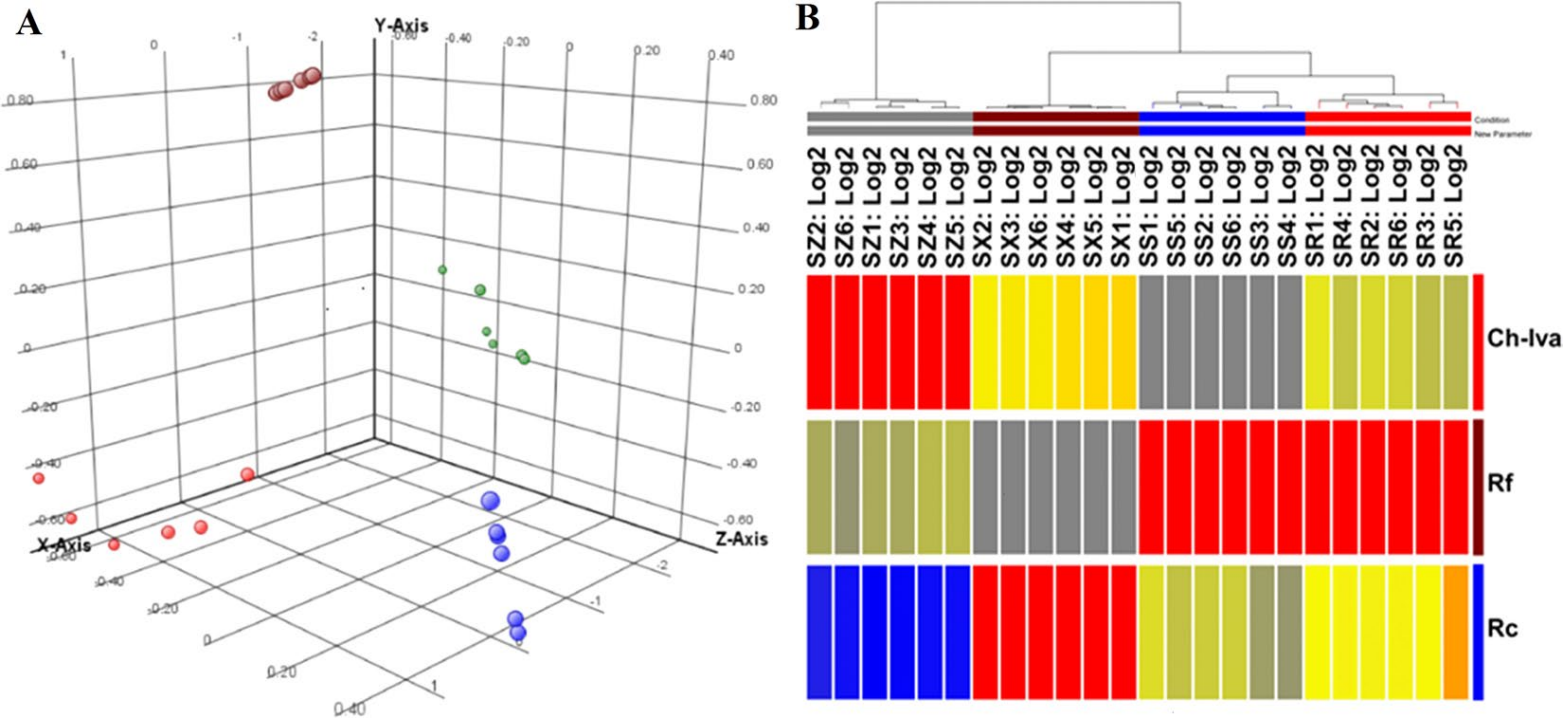
Four medicinal herbs were analyzed by principal component analysis ((**A**) samples of *Panax ginseng* are marked in light red, samples of *Panax notoginseng* are marked in blue, samples of *Panax quinquefolium* are marked in dark red, and samples of *Panax japlcus var* are marked in green) and clustering analysis ((**B**) SR1–SR6: *Panax ginseng*; SS1–SS6: *Panax notoginseng*; SX1–SX6: *Panax quinquefolium*; SZ1–SZ6: *Panax japlcus var*).

As a supplement to the verification, we randomly drew 6 samples from each type of medicinal herb in Table S1 and analyzed the samples using the three selected specific biomarkers through principal component analysis (PCA) as well as clustering analysis. The PCA was carried out, and the results showed that the four groups of samples were clearly separated (shown in Fig. 6A). Clustering analysis grouped these four groups of samples into four distinct clusters (shown in Fig. 6B), which was consistent with the result from the PCA analysis.

The quality control method and quantitative markers are recorded in the Pharmacopoeia of the People’s Republic of China (2010 version), which stipulates that the content of Re + Rg_1_ and Rb_1_ be not less than 0.30% and 0.20%, respectively. In our experiment, we investigated the peak areas of Re, Rg_1_ and Rb_1_ in the four types of samples, and identification by comparison with reference standards was usually required. Taking the peak areas of Re + Rg_1_ and Rb_1_ in the QC specimen as objects of reference, the ratios of the peak areas of Re + Rg_1_ and Rb_1_ in all samples to those in the QC specimen were analyzed (shown in Table S11). From Table S11, we found that the ratios of Re + Rg_1_ for *Panax ginseng*, *Panax notoginseng*, *Panax quinquefolium* and *Panax japlcus var* were 0.34–1.03, 1.05–1.42, 0.49–0.84, and 0.62–1.01, respectively, while the ratios of Rb_1_ for *Panax ginseng*, *Panax notoginseng*, *Panax quinquefolium* and *Panax japlcus var* were 0.36–1.32, 1.04–1.37, 1.03–1.29, and 0.65–1.17, respectively. The results suggested that the content of Re + Rg_1_ and Rb_1_ in some of the other three herbs was much higher than that in *Panax ginseng* and complied with the quantitative standard for *Panax ginseng*. Therefore, the traditional quantitative markers including Re, Rg_1_ and Rb_1_ cannot assure the quality of *Panax ginseng*, and it was difficult to differentiate *Panax ginseng* from the other three herbs using only the content of Re + Rg_1_ and Rb_1_. The traditional quantitative marker for *Panax ginseng* should be improved, and the results of our experiment remedied it. In fact, ginsenoside Rc and Rf are other major bioactive metabolites in *Panax ginseng* with large peak areas and high peak intensity, and the peak area of Ch-IVa takes up approximately one-sixth to one-fourth that of Rc. They are all easily detected and quantified.

## Conclusion

In this study, we conducted a metabolite fingerprinting analysis on a set of herbs that belong to the same genus and have extremely similar chemical metabolites to screen specific biomarkers from numerous metabolites in the designated herbs using metabolomics. The specific biomarkers were not easily chosen according to an intuitive comparison of their metabolite profiles but were chosen based on an analysis of the overall metabolites for each herb. The most specific respective biomarkers were obtained via multivariate statistical analysis and correlational analysis. Our results proved that metabolomics is a powerful technology platform for studying the specific biomarkers of medicinal herbs even though they are in the same genus, and our study provided a demonstration of the selection of specific biomarkers for other medicinal herbs. Moreover, this novel strategy can be used not only in the selection of specific biomarkers but also in the quality control of herbs and other compounds.

## Methods

### Medicinal herb materials

The major production areas of the four types of samples were studied, and groups of herbs were collected from different locations (shown in Table S1 and Table S2). Thirty-nine *Panax ginseng* samples (No. 1–6 and No. PG1–PG33) were collected from different production areas such as Jilin province, Heilongjiang province and Geumsan-gun, Korea. Fourteen *Panax notoginseng* samples (No. 7–12 and No. PN1-PN8) were obtained from Yunnan province and Guangxi province. Twenty-five *Panax quinquefolium* samples (No. 13–30 and No. PQ1–PQ7) were collected from Canada, America and China. Twenty-one *Panax japlcus var* samples (No. 31–42 and No. PJ1–PJ9) were collected from Shannxi province, Yunnan province, Sichuan province, Guizhou province, Gansu province and Hubei province. All samples were identified by Prof. Hong-bin Xiao, who is a co-author of this paper, and all voucher specimens were deposited in the Institute of Chinese Materia Medica, China Academy of Chinese Medical Sciences (Beijing, China). The 42 samples shown in Table S1 were used to screen and verify the specific biomarkers, while the other samples shown in Table S2 were used only to verify the specific biomarkers.

### Chemicals

Ultra-pure water was obtained from Honeywell International Inc. (Burdick & Jackson, Muskegon, MI, USA), and LC/MS-grade acetonitrile was purchased from J. T. Baker (Phillipsburg, NJ, USA). LC/MS-grade formic acid was obtained from Fisher Scientific (Fair Lawn, NJ, USA). The 7 total ginsenosides including chikusetsusaponin IVa (Ch-IVa, **R23**), ginsenoside Rf (**X21**), Rc (**Z11**), Re, Rg_1_ and Rb_1_ were either purchased from the National Institute for the Control of Pharmaceutical and Biological Products (Beijing, China) or were gifts from the State Key Laboratory of Natural and Biomimetic Drugs, Department of Natural Medicines, School of Pharmaceutical Sciences, Peking University. The purity of all of the reference standards was greater than 98%.

### Metabolite Extraction

Metabolite extractions were performed according to the references^19–21^. Each of the herbs was pulverized into powder (40 meshes). Each accurately weighed powder sample (1.0 g) was suspended in 20 mL of 70% aqueous MeOH and ultrasonically extracted (40 kHz, 200 W) for 30 min at 30 °C. The extracted solutions were then filtered. This extraction was repeated two additional times. The combined filtrate was evaporated to dryness using a rotary evaporator at 40 °C. The residue was dissolved in 5 mL of 70% aqueous MeOH, and the diluted solutions were filtered through a 0.22-µm nylon filter membrane prior to analysis.

### UPLC Conditions

The collected samples were analyzed on an Agilent 1290 UPLC coupled to a 6540 Q-TOF MS system with dual ESI source (Agilent Technologies, USA). All samples were separated on an Agilent ZORBAX RRHD Eclipse Plus C_18_ column (100 × 3 mm, 1.8 µm) connected to a Phenomenex Security Guard^TM^ ULTRA Cartridge using 0.1% formic acid-deionized water (A) and 0.1% formic acid-acetonitrile (B). The optimized gradient elution program was as follows: 0–7 min, 10–40% B; 7–9.5 min, 40–55% B; 9.5–12 min, 55–55% B; 12–15 min, 55–75% B. The temperature was set at 45 °C, and the injection volume was 1 µL. The data rate was set at 10 Hz, and the flow rate was 0.8 mL/min with a split ratio of 1:1. The wavelength was set at 203 nm.

### ESI Q-TOF MS Analysis

It has been proven^22^ that triterpenoid saponins are the major metabolites in the medicinal herbs of *Panax* genus, and they have a strong response in negative-ion mode. Therefore, the Agilent Q-TOF 6540 mass spectrometer (Agilent Technologies) was operated in negative-ion mode. The parameters of the ESI source were optimized as follows: gas temperature 300 °C, gas flow 5 L/min, nebulizer pressure 35 psi, sheath gas temperature 400 °C, sheath gas flow 12 L/min, capillary voltage 3500 V, nozzle voltage 1500 V, and fragmentor voltage 280 V. Internal references (Purine and HP-0921) were adopted to modify the measured masses in real time, and the reference masses in negative ion mode were at *m/z* 119.0363 and 1033.9881. The full scan range of the mass spectrometer was *m/z* 100–1700 for MS and MS/MS.

### Data processing

When selecting the specific biomarkers, all samples in Table S1 were analyzed by UPLC-Q-TOF MS to obtain their raw data. Then, the obtained UPLC Q-TOF MS raw data were further processed by Agilent Mass Hunter Qualitative Analysis software (version B.06.00, Agilent, America). The molecular feature extraction (MFE) algorithm was applied to extract metabolites from the total ion chromatograms (TICs) according to their metabolic features, including *m/z*, retention time and ion intensities, and the main parameters of MFE were optimized. The range of *m/z* values was 50 to 1700. Low-abundance ions can be hard to identify if the precursor ion intensity is low, generally below 5000 counts for an Agilent Q-TOF. To produce a matrix containing fewer biased and redundant data, the thresholds of peak filters and metabolite filters were set at 1000 counts and 5000 counts, respectively. All of the extracted metabolites were output to create a.cef file, which can be imported into Mass Profiler Professional (MPP) software (version B.12.00, Agilent) for further data processing. Alignment, normalization, defining the sample sets, filtering by frequency and Venn diagram analysis were applied to process the data. The metabolites with absolute abundance greater than 5000 counts were aligned by retention time and accurate mass; the tolerance windows of retention time and accurate mass were 0.08 min and 15 ppm, respectively. Missing peaks were filtered according to their frequency, and metabolites that appeared in 100% of samples in at least one group were retained. The final metabolites were subjected to Venn diagram analysis to select the specific biomarkers.

The correlations between the specific biomarkers were analyzed using partial correlational analysis. Their peak areas were recorded and imported into SPSS for Windows (version 16.0, Chicago, SPSS Inc.). The correlation networks were presented by Cytoscape software (Cytoscape 3.4.0).

## Electronic supplementary material

Screening Specific Biomarkers of Herbs Using a Metabolomics Approach: A Case Study of Panax ginseng
