# Supplementary material for: Screening Specific Biomarkers of Herbs Using a Metabolomics Approach: A Case Study of Panax ginseng
Source: Sci Rep. 2017 Jul 4;7:4609. doi: 10.1038/s41598-017-04712-7 (PMC5496890; doi:10.1038/s41598-017-04712-7)
Supplement: Supplementary file 1 — Screening Specific Biomarkers of Herbs Using a Metabolomics Approach: A Case Study of Panax ginseng [file 41598_2017_4712_MOESM1_ESM.pdf]

Screening Specific Biomarkers of Herbs Using a Metabolomics Approach: A Case Study of *Panax ginseng*

Hong-ping Wang [a], Yan Liu [b], Chang Chen [b], and Hong-bin Xiao [a,c]*

[a] Beijing University of Chinese Medicine, Beijing, China

[b] Institute of Chinese Materia Medica, China Academy of Chinese Medical Sciences, Beijing 100700, China

[c] Shihezi University, Shihezi, China

* Corresponding author: Tel.: +86 10 6248 6490; fax: +86 10 6248 6490.

E-mail address: hbxiao69@163.com

Supplementary Data

Table S1 List of production areas of *Panax* genus herbal materials used to screening the specific biomarkers.

No.	*Origins*
Panax ginseng	
1	Yangcha village, Taiwang town, Ji'an city, Jilin province
2	Xintunzi town, Fusong county, Jilin province
3	Qinggouzi town, Dunhua city, Jilin province
4	Fuxing town, Wangqing county, Jilin province
5	Mengjiang town, Jingyu county, Jilin province
6	Liaohuangdi Baoquanshan, Changbai county, Jilin province
Panax notoginseng	
7	Songshuping village, Zhuilijie town, Wenshan county, Wenshan state, Yunnan province, China
8	Yiwu, Leshichong town, Wenshan county, Wenshan state, Yunnan province, China
9	Laowuji, Leshichong town, Wenshan county, Wenshan state, Yunnan province, China
10	Leshichong town, Wenshan county, Wenshan state, Yunnan province, China
11	Huangcaoba village, Pingba town, Wenshan county, Wenshan state, Yunnan province, China
12	Laojunshan village, Xiaojie town, Wenshan county, Wenshan state, Yunnan province, China
Panax quinquefolium	
13	Western Ontario, Canada
14	Western Ontario, Canada
15	Western Ontario, Canada
16	Western Ontario, Canada
17	Western Ontario, Canada
18	Western Ontario, Canada
19	Wisconsin, USA
20	Wisconsin, USA
21	Wisconsin, USA
22	Wisconsin, USA
23	Wisconsin, USA
24	Wisconsin, USA
25	Changbai Mountain, Jilin province, China
26	Changbai Mountain, Jilin province, China
27	Changbai Mountain, Jilin province, China
28	Changbai Mountain, Jilin province, China
29	Changbai Mountain, Jilin province, China
30	Changbai Mountain, Jilin province, China
Panax japlcus var	
31	Taibai county, Baoji city, Shannxi province, China

32	Taibai county, Baoji city, Shannxi province, China
33	Taibai county, Baoji city, Shannxi province, China
34	Mei county, Baoji city, Shannxi province, China
35	Mei county, Baoji city, Shannxi province, China
36	Mei county, Baoji city, Shannxi province, China
37	Lanping county, Nujiang state, Yunnan province, China
38	Lanping county, Nujiang state, Yunnan province, China
39	Lanping county, Nujiang state, Yunnan province, China
40	Lanping county, Nujiang state, Yunnan province, China
41	Heqing county, Dali state, Yunnan province, China
42	Eryuan county, Dali state, Yunnan province, China

Table S2 The *Panax* genus herbal materials participating in the verification of the specific biomarkers.

No.	*Origins*
Panax ginseng	
PG1	Zhengyi village, Dalu town, Ji'an city, Jilin province, China
PG2	Yangzigou village, Yulin town, Ji'an city, Jilin province, China
PG3	Haozigou village, Qingshi town, Ji'an city, Jilin province, China
PG4	Mati village, Caiyuan town, Ji'an city, Jilin province, China
PG5	Louzigou village, Toudao town, Ji'an city, Jilin province, China
PG6	Beigang, Fusong county, Jilin province, China
PG7	Xintunzi town, Fusong county, Jilin province, China
PG8	Quanyang Jiangdong village, Fusong county, Jilin province, China
PG9	Lushuihe Shalizihe village, Fusong county, Jilin province, China
PG10	Jianghe town, Fusong county, Jilin province, China
PG11	Dafang village, Wanliang town, Fusong county, Jilin province, China
PG12	Hanzhang town, Dunhua city, Jilin province, China
PG13	Shangri village, Dunhua city, Jilin province, China
PG14	Tianqiaoling Zhuanping, Wangqing county, Jilin province, China
PG15	Xihe, Luozigou town, Wangqing county, Jilin province, China
PG16	Fuxing village, Mengjiang town, Jingyu county, Jilin province, China
PG17	Zhuanshanzi, Mengjiang town, Jingyu county, Jilin province, China
PG18	Xujiadian village, Jingyu county, Jilin province, China
PG19	Sisea, Jingyu county, Jilin province, China
PG20	Xinhe, Antu county, Jilin province, China
PG21	Liangjiang town, Antu county, Jilin province, China
PG22	Xinfangzi town, Changbai county, Jilin province, China
PG23	Erdaogang village, Changbai county, Jilin province, China
PG24	Dadingzi village, Xinfangzi town, Changbai county, Jilin province, China
PG25	Dongxiaoshan farm, Linjiang city, Jilin province, China
PG26	Dongbeicha, Linjiang city, Jilin province, China
PG27	Piaohe town, Jiaohe city, Jilin province, China
PG28	Changling farm, Qianjin town, Jiaohe city, Jilin province, China
PG29	Qitaihe city, Heilongjiang province, China
PG30	Gonghe town, Mudanjiang city, Heilongjiang province, China
PG31	Qingan forestry bureau Jingou forest farm, Tieli city, Heilongjiang province, China
PG32	Geumsan-gun, Korean
PG33	Geumsan-gun, Korean
Panax notoginseng	
PN1	Baxin town, Wenshan county, Wenshan state, Yunnan province, China
PN2	Matang town, Wenshan county, Wenshan state, Yunnan province, China
PN3	Bazhai town, Maguan county, Wenshan state, Yunnan province, China

PN4	Zhela town, Yanshan county, Wenshan state, Yunnan province, China
PN5	Panlong town, Yanshan county, Wenshan state, Yunnan province, China
PN6	Badaoshao town, Qiubei county, Wenshan state, Yunnan province, China
PN7	Laozhai town, Mengzi county, Honghe state, Yunnan province, China
PN8	Sanhe town, Jingxi county, Bose city, Guangxi province, China
Panax quinquefolium	
PQ1	Jingyu county, Jilin province, China
PQ2	Ji'an city, Jilin province, China
PQ3	Tonghua county, Jilin province, China
PQ4	Dunhua county, Jilin province, China
PQ5	Linjiang city, Jilin province, China
PQ6	Heilongjiang province, China
PQ7	Huairou district, Beijing city, China
Panax japlcus var	
PJ1	Ningqiang county, Hanzhong city, Shannxi province, China
PJ2	Ningqiang county, Hanzhong city, Shannxi province, China
PJ3	Ningqiang county, Hanzhong city, Shannxi province, China
PJ4	Maguan county, Wenshan state, Yunnan province, China
PJ5	Chengjiang county, Yuxi city, Yunnan province, China
PJ6	Chendu city, Sichuan province, China
PJ7	Xingyi city, Guizhou province, China
PJ8	Wen county, Longnan city, Gansu province, China
PJ9	Enshi city, Hubei province, China

Table S3 The shifts of retention time, *m/z*, peak areas and intensity in 15 sets of data obtained via acquisition of QC specimen.

**Rt (min)**	***m/z***	**Diff (ppm)**	***RSD* (Rt)**	***RSD* (peak areas)**	***RSD* (peak intensity)**
**4.58**	931.5266	1.61-2.47	0.13%	6.02%	1.66%
**6.76**	799.4844	0.13-2.63	0.05%	7.95%	1.27%
**7.52**	1077.5850	0.09-1.39	0.04%	8.54%	3.45%
**8.07**	945.5423	0.21-2.43	0.05%	3.66%	2.47%
**9.52**	793.4374	0.76-2.52	0.03%	5.19%	2.97%

Table S4 The 26 specific metabolites presented in A for A versus B.

No.	*m/z*	Rt(min)	Compounds	No.	*m/z*	Rt(min)	Compounds
R1	1013.5270	4.89	-	R14	935.5117	7.30	-
R2	1013.5270	8.07	-	R15	1494.7890	7.31	-
R3	867.4675	4.93	-	R16	1495.2910	7.31	-
R4	1031.5440	5.37	-	R17	1434.2430	7.32	-
R5	1117.5440	6.29	-	R18	1433.7420	7.32	-
R6	862.4814	6.76	-	R19	1018.4870	7.32	-
R7	835.4623	6.76	-	R20	1163.5870	7.39	Ma-Rc
R8	1661.8980	7.05	-	R21	1163.5860	7.75	Ma-Rb_3
R9	1113.5630	7.29	-	R22	925.4807	7.67	-
R10	1113.5630	7.54	-	R23	793.4388	8.09	Ch-Ⅳa
R11	1140.5820	7.29	-	R24	793.4382	9.52	-
R12	1140.5820	7.54	-	R25	677.3745	11.00	-
R13	936.0135	7.30	-	R26	677.3702	11.41	-

Table S5 The 23 specific metabolites presented in A for A versus C.

No.	*m/z*	Rt(min)	Compounds	No.	*m/z*	Rt(min)	Compounds
X1	931.5237	4.49	Notoginsenoside R_1 isomer	X13	1494.7890	7.31	-
X2	1013.5270	4.89	-	X14	1495.2910	7.31	-
X3	1013.5270	8.07	-	X15	1163.5860	7.75	Ma-Rb_3
X4	1043.5120	4.89	-	X16	790.5811	4.51	-
X5	862.4814	6.75	-	X17	853.5768	4.51	-
X6	835.4623	6.76	-	X18	903.6652	4.81	-
X7	841.4961	5.20	Acetyl-Rg_1	X19	781.4753	5.20	-
X8	841.4962	5.46	Acetyl-Rg_1 isomer	X20	799.4855	5.23	Ia
X9	841.4960	6.82	Acetyl-Rf	X21	799.4842	6.62	Rf
X10	1661.8980	7.05	-	X22	1089.5860	7.14	-
X11	1113.5630	7.54	-	X23	981.5198	8.07	-
X12	1140.5820	7.54	-				

Table S6 The 30 specific metabolites presented in A for A versus D.

No.	*m/z*	Rt(min)	Compounds	No.	*m/z*	Rt(min)	Compounds
Z1	931.5273	4.36	Re_4	Z16	1495.2910	7.31	-
Z2	1013.5270	8.07	-	Z17	1434.2430	7.32	-
Z3	1013.5270	4.89	-	Z18	1433.7420	7.32	-
Z4	867.4675	4.93	-	Z19	1018.4870	7.32	-
Z5	1031.5440	5.37	-	Z20	1163.5870	7.39	Ma-Rc
Z6	1117.5440	6.29	-	Z21	1163.5860	7.75	-
Z7	862.4814	6.75	-	Z22	925.4807	7.67	-
Z8	835.4623	6.75	-	Z23	925.4811	7.76	-
Z9	1113.5630	7.29	-	Z24	677.3745	11.00	-
Z10	1113.5630	7.54	-	Z25	1008.5390	8.07	-
Z11	1077.5860	7.29	Rc	Z26	790.5811	4.51	-
Z12	1140.5820	7.29	-	Z27	853.5768	4.51	-
Z13	936.0135	7.30	-	Z28	903.6652	4.81	-
Z14	935.5117	7.30	-	Z29	781.4753	5.20	-
Z15	1494.7890	7.31	-	Z30	981.5198	8.07	-

Table S7 The metabolites identified in *Panax ginseng*.

No.	t_R (min)	Molecular formula	Measured value (m/z)	Diff (ppm)	Product ions	Compound name
Z1	4.36	$C_{47}H_{80}O_{18}$	931.4966	4.6	799.3830[M-H-Ara(f)]$^-$, 637.3525[M-H-Ara(f)-Glc]$^-$, 475.3223[M-H-Ara(f)-2Glc]$^-$	Re_4
X1	4.49	$C_{47}H_{80}O_{18}$	931.5255	-1.2	799.4899[M-H-Xyl]$^-$, 637.4287[M-H-Xyl-Glc]$^-$, 475.3819[M-H-Xyl-2Glc]$^-$	Notoginsenoside R_1 isomer
X7	5.20	$C_{44}H_{74}O_{15}$	841.4960	0.6	799.4901[M-H-Ac]$^-$, 637.4284[M-H-Ac-Glc]$^-$, 475.3797[M-H-Ac-2Glc]$^-$	Acetyl-Rg_1
X20	5.23	$C_{42}H_{72}O_{14}$	799.4846	-0.4	637.4335[M-H-Glc]$^-$, 475.3803[M-H-2Glc]$^-$	Ia
X8	5.46	$C_{44}H_{74}O_{15}$	841.4957	0.2	637.4340[M-H-Ac-Glc]$^-$, 475.3801[M-H-Ac-2Glc]$^-$	Acetyl-Rg_1 isomer
X21	6.62	$C_{42}H_{72}O_{14}$	799.4850	1.3	637.4254[M-H-Glc]$^-$, 475.3825[M-H-2Glc]$^-$	Rf
X9	6.82	$C_{44}H_{74}O_{15}$	841.4936	-1.5	799.4907[M-H-Ac]$^-$, 637.4289[M-H-Ac-Glc]$^-$, 475.3792[M-H-Ac-2Glc]$^-$	Acetyl-Rf
Z11	7.29	$C_{53}H_{90}O_{22}$	1077.5853	2.5	945.5445[M-H-Ara(f)]$^-$, 783.4902[M-H-Ara(f)-Glc]$^-$, 621.4376 [M-H-Ara(f)-2Glc]$^-$, 459.3851[M-H-Ara(f)-3Glc]$^-$	Rc
R20/Z20	7.39	$C_{56}H_{92}O_{25}$	1163.5843	-0.5	1119.6102[M-H-CO_2]$^-$, 1077.5867[M-H-CO_2-Ac]$^-$, 945.5449[M-H-CO_2-Ac-Ara(f)]$^-$, 783.4891[M-H-CO_2-Ac-Ara(f)-Glc]$^-$, 621.4385[M-H-CO_2-Ac-Ara(f)-2Glc]$^-$, 459.3851[M-H-CO_2-Ac-Ara(f)-3Glc]$^-$	Malonyl-ginsenoside Rc
R21/X15	7.75	$C_{56}H_{92}O_{25}$	1163.5836	-1.1	1119.6035[M-H-CO_2]$^-$, 1077.5927[M-H-CO_2-Ac]$^-$, 945.5463[M-H-CO_2-Ac-Xyl]$^-$, 783.4899[M-H-CO_2-Ac-Xyl-Glc]$^-$, 621.4405[M-H-CO_2-Ac-Xyl-2Glc]$^-$, 459.3893[M-H-CO_2-Ac-Xyl-3Glc]$^-$	Malonyl-ginsenoside Rb_3
R23	8.09	$C_{42}H_{66}O_{14}$	793.4388	1.0	631.3853[M-H-Glc]$^-$, 455.3532[M-H-Glc-Glu A]$^-$	Ch-IVa

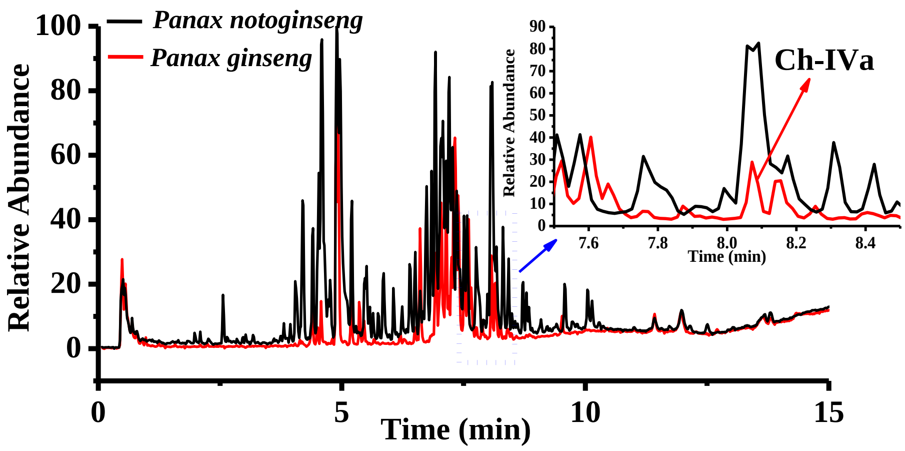

Fig. S1 The overlapped metabolite profiling of *Panax ginseng* and *Panax notoginseng*.

Table S8 The peak areas of Ch-IVa in *Panax ginseng* and *Panax notoginseng*.

Panax ginseng			*Panax notoginseng*		
Sample numbers	Peak areas	*RSD* (%)	Sample numbers	Peak areas	*RSD* (%)
1	102737.43	15.83	7	1145.09	13.45
2	105273.78		8	1151.09	
3	92630.02		9	1120.19	
4	97138.21		10	1479.11	
5	90649.96		11	1269.96	
6	110386.48		12	1305.33	
PG1	125855.89		PN1	1501.12	
PG2	110886.58		PN2	1088.77	
PG3	99415.38		PN3	1522.98	
PG4	118759.01		PN4	1499.16	
PG5	154223.75		PN5	1311.69	
PG6	117363.72		PN6	1415.87	
PG7	97363.82		PN7	1623.29	
PG8	103736.23		PN8	1577.21	
PG9	126393.29				
PG10	101736.73				
PG11	142233.98				
PG12	127367.93				
PG13	159273.38				
PG14	153623.83				
PG15	119263.92				
PG16	132623.28				
PG17	97263.72				
PG18	123934.19				
PG19	132827.32				
PG20	119373.76				
PG21	142373.65				
PG22	102836.48				
PG23	138263.83				
PG24	92873.63				
PG25	99725.53				
PG26	107263.92				
PG27	129983.63				
PG28	136538.21				
PG29	119364.45				
PG30	127339.61				
PG31	136754.82				
PG32	108862.76				
PG33	98531.94				

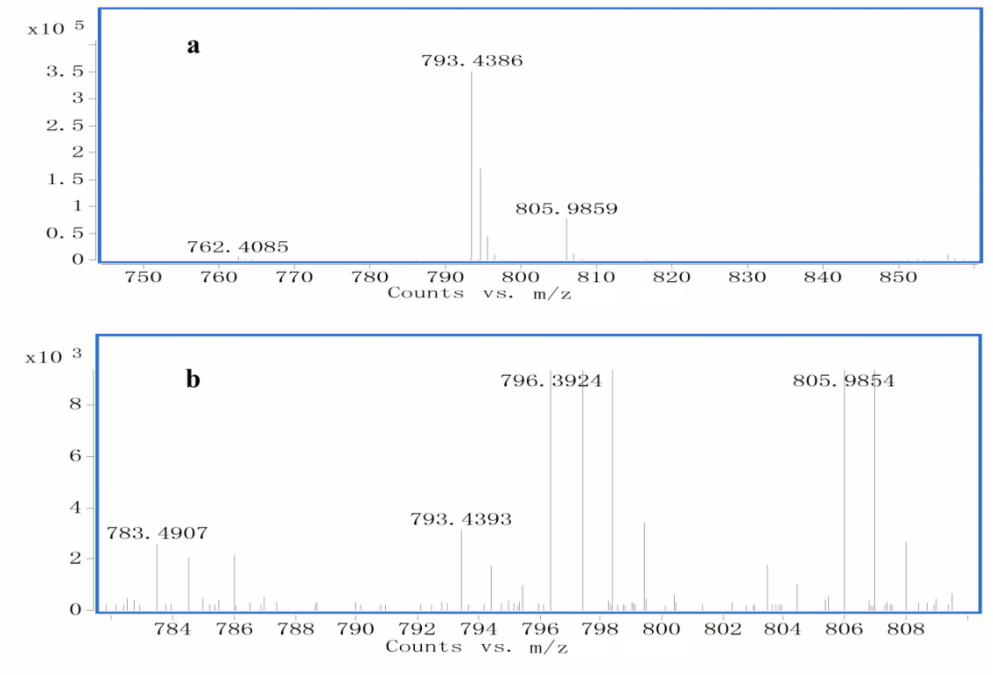

Fig. S2 The MS spectrum of Ch-IVa in *Panax ginseng* (a) and *Panax notoginseng* (b): the absolute abundance of its precursor ion was much higher in *Panax ginseng* (a) and lower than 5000 counts in *Panax notoginseng* (b).

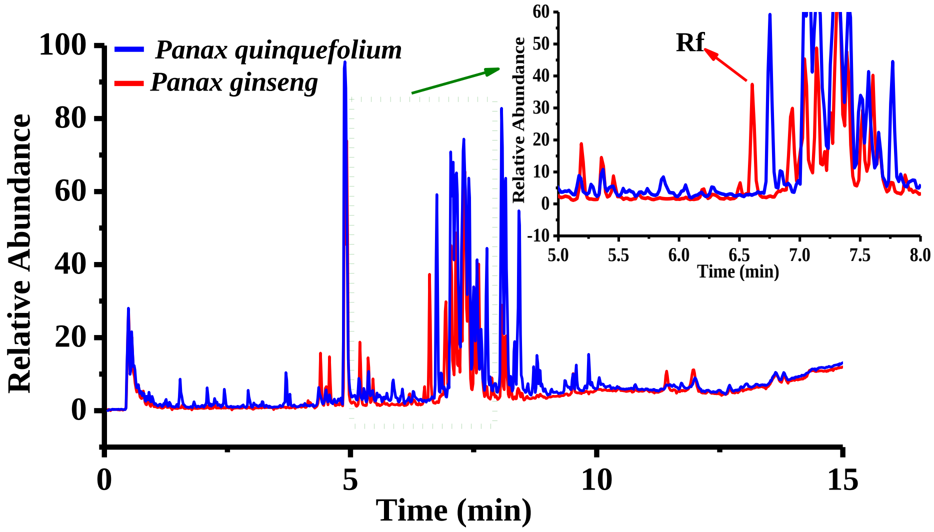

Fig. S3 The overlapped metabolite profiling of *Panax ginseng* and *Panax quinquefolium*.

Table S9 The peak areas of ginsenoside Rf in *Panax ginseng* and *Panax quinquefolium*.

Panax ginseng			*Panax quinquefolium*		
Sample numbers	Peak areas	*RSD* (%)	Sample numbers	Peak areas	*RSD* (%)
1	1144518.88	14.80	13	276.19	11.36
2	1101032.84		14	225.69	
3	1114872.19		15	240.01	
4	1269263.61		16	223.15	
5	1011383.27		17	203.72	
6	1143370.04		18	206.07	
PG1	1080240.21		19	203.62	
PG2	1092738.85		20	207.73	
PG3	1151661.05		21	210.38	
PG4	1358781.48		22	251.48	
PG5	1580186.09		23	258.11	
PG6	1190942.51		24	272.01	
PG7	1190942.51		25	232.19	
PG8	1444486.49		26	200.72	
PG9	1473054.83		27	231.29	
PG10	1478411.39		28	199.71	
PG11	942755.09		29	213.96	
PG12	1449843.05		30	250.82	
PG13	837409.35		PQ1	271.22	
PG14	1280218.56		PQ2	252.18	
PG15	1512336.29		PQ3	221.34	
PG16	1330213.15		PQ4	280.89	
PG17	1421274.72		PQ5	208.47	
PG18	1089167.81		PQ6	263.22	
PG19	1385564.30		PQ7	248.19	
PG20	983822.07				
PG21	1456985.14				
PG22	1323071.06				
PG23	1019532.49				
PG24	1117736.15				
PG25	1290931.68				
PG26	1551617.75				
PG27	1164159.69				
PG28	1410561.59				
PG29	1369494.61				
PG30	1244508.14				
PG31	1376636.69				
PG32	1498052.12				
PG33	1169516.26				

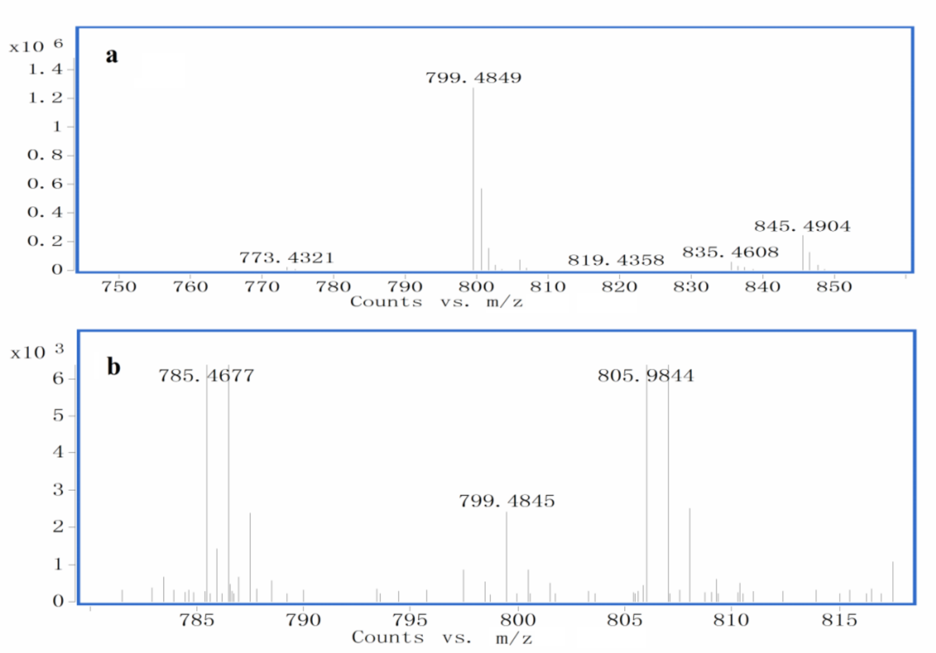

Fig. S4 The MS spectrum of ginsenoside Rf in *Panax ginseng* (a) and *Panax quinquefolium* (b): the absolute abundance of its precursor ion was much higher in *Panax ginseng* (a) and lower than 5000 counts in *Panax quinquefolium* (b).

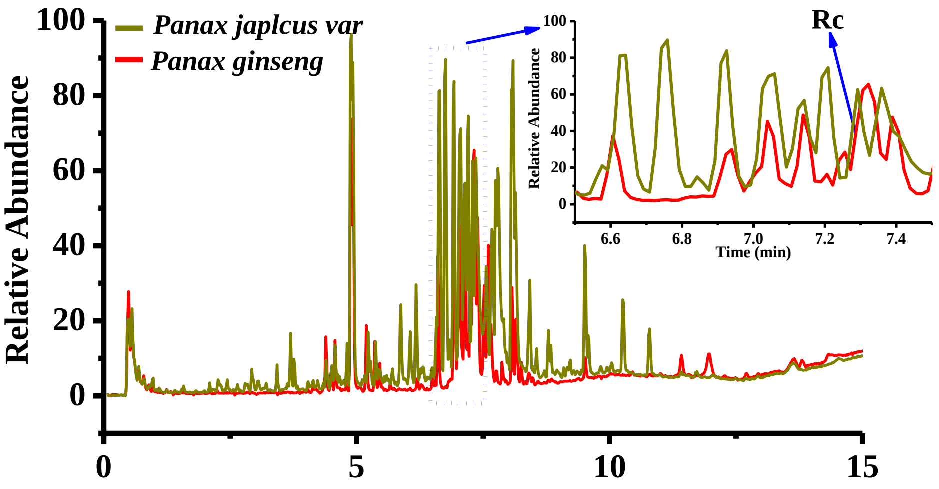

Fig. S5 The overlapped metabolite profiling of *Panax ginseng* and *Panax japlcus var*.

Table S10 The peak areas of ginsenoside Rc in *Panax ginseng* and *Panax japlcus var*.

Panax ginseng			*Panax japlcus var*		
Sample numbers	Peak areas	*RSD* (%)	Sample numbers	Peak areas	*RSD* (%)
1	436505.47	19.31	31	1986.23	10.44
2	447581.19		32	1801.15	
3	482460.22		33	2198.91	
4	503663.86		34	2406.45	
5	451339.24		35	2137.07	
6	434072.03		36	2079.03	
PG1	636811.73		37	1915.01	
PG2	587024.63		38	1871.23	
PG3	578919.75		39	1700.29	
PG4	681581.52		40	1818.64	
PG5	597059.24		41	2104.85	
PG6	744876.75		42	2267.63	
PG7	807786.02		PJ1	2388.14	
PG8	776138.41		PJ2	2205.19	
PG9	675406.38		PJ3	2286.24	
PG10	639899.30		PJ4	2033.59	
PG11	813575.22		PJ5	1822.97	
PG12	697405.33		PJ6	2401.85	
PG13	466995.27		PJ7	2133.94	
PG14	443452.53		PJ8	2371.45	
PG15	766103.80		PJ9	1968.77	
PG16	792734.11				
PG17	835574.17				
PG18	720948.06				
PG19	848696.35				
PG20	602076.54				
PG21	773436.79				
PG22	734456.19				
PG23	580463.54				
PG24	558464.59				
PG25	668459.34				
PG26	703194.52				
PG27	491695.84				
PG28	673862.59				
PG29	716316.70				
PG30	671546.91				
PG31	695089.65				
PG32	730210.78				
PG33	592813.82				

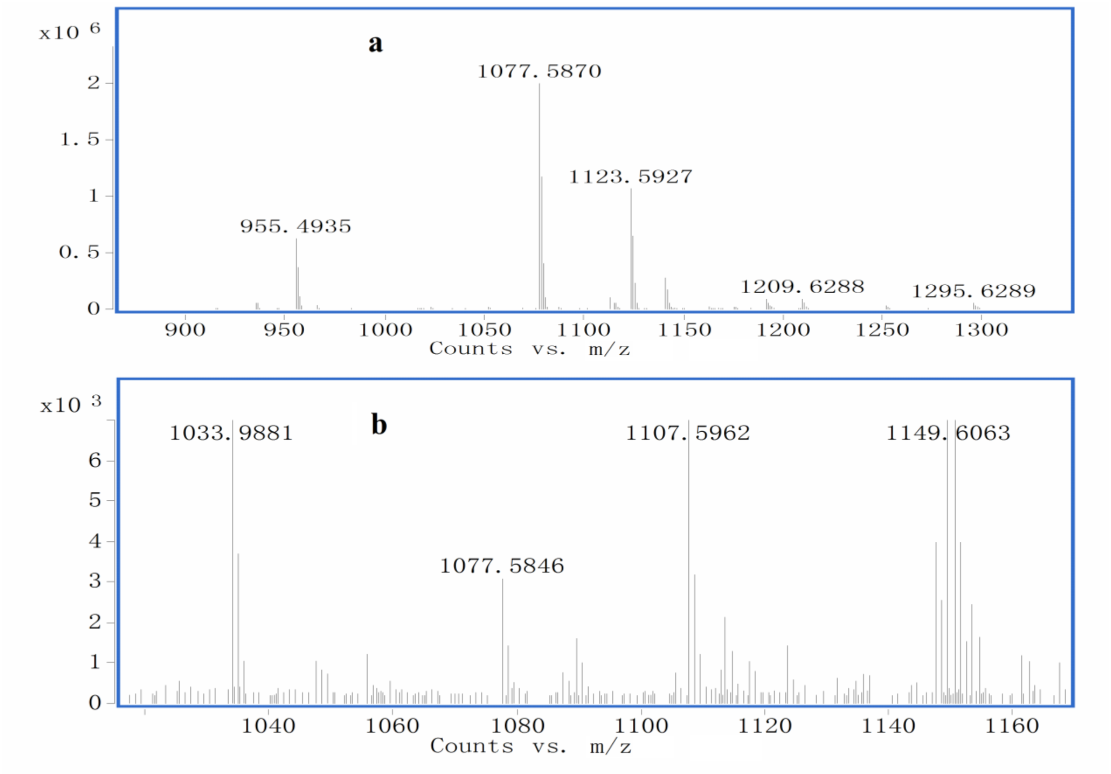

Fig. S4 The MS spectrum of ginsenoside Rf in *Panax ginseng* (a) and *Panax japlcus var* (b): the absolute abundance of its precursor ion was much higher in *Panax ginseng* (a) and lower than 5000 counts in *Panax japlcus var* (b).

Table S11 The peak areas of Re + Rg_1, Rb_1 in samples and QC specimen and the ratios of peak areas of Re + Rg_1, Rb_1 in samples to those in QC specimen, respectively.

Herbal materials	Sample numbers	Peak areas of Re + Rg_1	Peak areas of Rb_1	The ratios of peak areas of Re + Rg_1 ^{a}	The ratios of peak areas of Rb_1 ^{b}
QC specimen	-	5054879.23	2040566.05	1.00	1.00
Panax ginseng	1	2990148.75	1041927.85	0.59	0.51
	2	3458890.27	1379563.79	0.68	0.68
	3	3260270.88	1330547.38	0.64	0.65
	4	4009319.72	1448812.12	0.79	0.71
	5	2397973.85	1069389.70	0.47	0.52
	6	4173721.31	1729484.72	0.83	0.85
	PG1	2196884.79	1202496.65	0.43	0.59
	PG2	2461769.91	2215192.46	0.49	1.09
	PG3	3591708.27	1224151.78	0.71	0.60
	PG4	3521022.02	1838775.34	0.70	0.90
	PG5	3525907.09	2171245.28	0.70	1.06
	PG6	3860472.93	2017748.62	0.76	0.99
	PG7	2805817.54	2072523.36	0.56	1.02
	PG8	3404899.73	1796738.91	0.67	0.88
	PG9	3897790.39	1629867.02	0.77	0.80
	PG10	3096235.32	1689737.09	0.61	0.83
	PG11	3652478.97	2690331.49	0.72	1.32
	PG12	2434211.64	2061058.88	0.48	1.01
	PG13	2767731.97	1150906.49	0.55	0.56
	PG14	3267935.66	1364910.13	0.65	0.67
	PG15	4006480.70	2502441.39	0.79	1.23
	PG16	2991488.43	1367457.79	0.59	0.67
	PG17	2419297.45	1477644.19	0.48	0.72
	PG18	2699780.46	1596747.41	0.53	0.78
	PG19	4988815.10	2151500.90	0.99	1.05
	PG20	2395837.73	727357.62	0.47	0.36
	PG21	3859805.66	2492887.66	0.76	1.22
	PG22	3436531.84	2434291.42	0.68	1.19
	PG23	2716131.61	1626045.53	0.54	0.80
	PG24	2669555.92	1866162.71	0.53	0.91
	PG25	3846776.89	1857245.89	0.76	0.91
	PG26	5226102.99	2369962.95	1.03	1.16
	PG27	1740046.41	756018.82	0.34	0.37
	PG28	3308388.90	2462952.63	0.65	1.21
	PG29	3142086.56	1563627.80	0.62	0.77
	PG30	2568186.81	1092310.25	0.51	0.54

	PG31	2966482.44	2323468.11	0.59	1.14
	PG32	2833361.17	2182709.76	0.56	1.07
	PG33	2458092.72	1016517.30	0.49	0.50
Panax notoginseng	7	7196048.76	2535466.28	1.42	1.24
	8	6546932.25	2334509.36	1.30	1.14
	9	5781888.66	2557936.21	1.14	1.25
	10	6649239.54	2397748.74	1.32	1.18
	11	5726017.24	2474571.92	1.13	1.21
	12	5982517.17	2797542.32	1.18	1.37
	PN1	5350511.21	2130273.34	1.06	1.04
	PN2	5741003.50	2237299.69	1.14	1.10
	PN3	6340743.50	2403720.57	1.25	1.18
	PN4	5323409.57	2203720.29	1.05	1.08
	PN5	5357672.76	2139273.72	1.06	1.05
	PN6	5623959.61	2203782.53	1.11	1.08
	PN7	6756619.61	2420376.87	1.34	1.19
	PN8	6049635.21	2320720.35	1.20	1.14
Panax quinquefolium	13	3360704.12	2596969.95	0.66	1.27
	14	3386613.77	2594248.69	0.67	1.27
	15	3348000.20	2590018.20	0.66	1.27
	16	3338651.79	2507983.78	0.66	1.23
	17	3308254.42	2306029.20	0.65	1.13
	18	3378096.32	2577088.90	0.67	1.26
	19	3087958.05	2103601.57	0.61	1.03
	20	3093264.15	2509503.67	0.61	1.23
	21	2960487.04	2552502.49	0.59	1.25
	22	4181833.98	2258664.10	0.83	1.11
	23	4248141.03	2524000.50	0.84	1.24
	24	4008572.37	2426141.82	0.79	1.19
	25	2849767.34	2301070.32	0.56	1.13
	26	2767352.62	2266429.99	0.55	1.11
	27	2844370.51	2254861.76	0.56	1.11
	28	3012487.86	2225703.46	0.60	1.09
	29	2890710.46	2380887.97	0.57	1.17
	30	2480903.54	2210823.30	0.49	1.08
	PQ1	3022047.90	2285736.93	0.60	1.12
	PQ2	3116665.53	2638268.52	0.62	1.29
	PQ3	3222010.63	2629377.38	0.64	1.29
	PQ4	2967020.00	2392837.82	0.59	1.17
	PQ5	3407570.65	2502730.02	0.67	1.23
	PQ6	3167736.23	2530727.96	0.63	1.24
	PQ7	2820913.11	2302374.29	0.56	1.13
	31	4496957.21	1349061.88	0.89	0.66

Panax japlcus var	32	4487802.45	1419880.90	0.89	0.70
	33	4311593.89	1432761.74	0.85	0.70
	34	4396161.87	1367256.75	0.87	0.67
	35	4277577.74	1350959.37	0.85	0.66
	36	4384160.24	1323406.39	0.87	0.65
	37	5099361.57	2228717.31	1.01	1.09
	38	5131705.86	2244538.13	1.02	1.10
	39	4799192.57	2173732.03	0.95	1.07
	40	5061487.80	2205361.53	1.00	1.08
	41	4935899.36	2204961.04	0.98	1.08
	42	5043792.28	2190568.69	1.00	1.07
	PJ1	4433667.08	1577328.37	0.88	0.77
	PJ2	4437604.52	1537152.23	0.88	0.75
	PJ3	4403700.33	1593372.46	0.87	0.78
	PJ4	4474613.35	2106283.19	0.89	1.03
	PJ5	4389094.49	1937461.44	0.87	0.95
	PJ6	4085177.64	1836493.31	0.81	0.90
	PJ7	3927478.61	1633726.65	0.78	0.80
	PJ8	4991592.63	2383642.49	0.99	1.17
	PJ9	3129936.31	1975395.71	0.62	0.97

a: The ratios of peak areas of Re + Rg_1 in samples to that in QC specimen.

b: The ratios of peak areas of Rb_1 in samples to that in QC specimen.
